# Global positioning system survey data for active seismic and volcanic areas of eastern Sicily, 1994 to 2013

**DOI:** 10.1038/sdata.2016.62

**Published:** 2016-08-01

**Authors:** Alessandro Bonforte, Sonia Fagone, Carmelo Giardina, Simone Genovese, Gianpiero Aiesi, Francesco Calvagna, Massimo Cantarero, Orazio Consoli, Salvatore Consoli, Francesco Guglielmino, Biagio Puglisi, Giuseppe Puglisi, Benedetto Saraceno

**Affiliations:** 1 Istituto Nazionale di Geofisica e Vulcanologia, Sezione di Catania—Osservatorio Etneo. Piazza Roma, 2–95125 Catania, Italy; 2 Geologist, external cooperator, etna2000.com, Freelance Geologist, 95100 Catania, Italy

**Keywords:** Geophysics, Natural hazards, Tectonics, Volcanology, Geodynamics

## Abstract

This work presents and describes a 20-year long database of GPS data collected by geodetic surveys over the seismically and volcanically active eastern Sicily, for a total of more than 6300 measurements. Raw data were initially collected from the various archives at the Istituto Nazionale di Geofisica e Vulcanologia, Sezione di Catania—Osservatorio Etneo and organized in a single repository. Here, quality and completeness checks were performed, while all necessary supplementary information were searched, collected, validated and organized together with the relevant data. Once all data and information collections were completed, raw binary data were converted into the universal ASCII RINEX format; all data are provided in this format with the necessary information for precise processing. In order to make the data archive readily consultable, we developed software allowing the user to easily search and obtain the needed data by simple alphanumeric and geographic queries.

## Background & Summary

The focus of this work is to present and describe the database of GPS data collected during several geodetic surveys carried out on the Sicilian geodetic networks on volcanic and seismic areas from 1994 to 2013 (refs 1–4). Several changes were made over this period: the instrumentation was improved according to technological innovation; the networks configuration was continuously updated, by installing new benchmarks or replacing the old ones, in order to optimize logistics, reduce the workload and increase the spatial resolution. The benchmarks have been progressively improved and standardized by installing self-centering standard pins, in order to avoid station set-up errors (e.g. different instrumental height measurements). To make using such a large and heterogeneous data set easier, it was necessary to spend time and effort on collecting the necessary metadata (i.e. the information about each measurement, such as instrumentation, antenna height and so on, either digitally- or physically-supported) and check its completeness, to make an inventory of the available data and store it in a single and readable form.

The following strategy was adopted to achieve this goal. First, we adopted the RINEX (Receiver INdependent EXchange) format, the universal standard data interchange format for raw satellite navigation system data^5^, to archive GPS data. Second, we stored and verified all the relevant supplementary information collected in the field over the years, which has been organized in tables, for each survey and measurement session. These tables contain the metadata on the GPS data we intend to organize that allow translating the raw (proprietary) GPS data into the RINEX format. Each RINEX file thus contains a complete header with all necessary information on how the data were collected allowing any user to use this data.

The aim of this work was to make this valuable RINEX data archive easily accessible to all, by organizing and distributing an interactive database, where the data is readily discoverable by appropriate queries based on the time, location or instrumentation. A valuable dataset on active seismic and volcanic areas is thus provided, collected over a unique period of more than 20 years, making it suitable to investigate a wide range of geodynamic phenomena, from a single fault, earthquake or fast and local dyke intrusion to the regional tectonics or wide and long-lasting dynamics of a volcano^6–15^. With this database we want to open all the data collected by surveys carried out until 2013 in a single repository; new geodetic data are continuously being collected, especially by permanent networks, and the new GNSS data will be published soon on a dedicated database on the INGV website, according to the INGV data policy.

## Methods

### GPS networks in the Sicilian area

**Figure 1:**
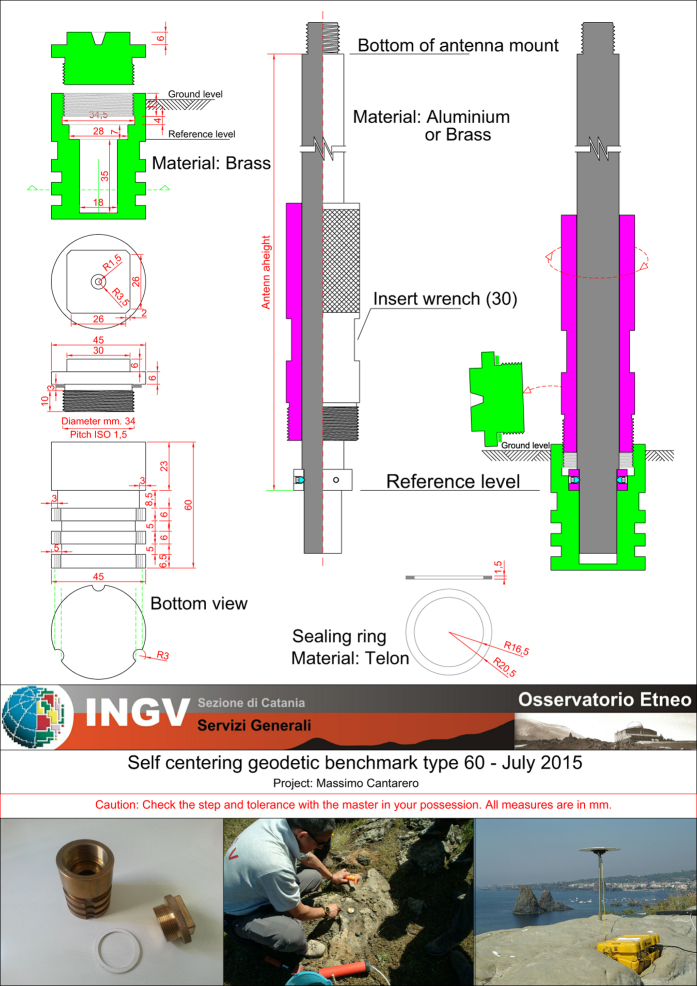
Detailed scheme of a self-centering benchmark.

The ground deformation monitoring of the volcanic and seismic areas in Eastern Sicily began in the mid-70s in the framework of the activities of the ‘Istituto Internazionale di Vulcanologia’ (IIV) of the Italian National Research Council (CNR), becoming in 2000 the Catania Section of the ‘Istituto Nazionale di Geofisica e Vulcanologia’ (INGV). The first geodetic network for monitoring purposes, surveyed by using Electronic Distance Meters (EDM), was installed by the IIV on the Vulcano and Lipari islands in 1975. In 1979, similar networks were also installed on Pantelleria and on the NE flank of Mt. Etna, followed by two other networks on the western and southern sides of Mt. Etna in 1980 and 1983, respectively. These earliest networks were surveyed until the 1990s by carrying out very accurate trilateration measurements using EDM, a cutting-edge technique for these years, often coupled with other precise topographic techniques^16,17^; due to the very high precision, leveling is still used for accurate vertical ground deformation measurements^18,19^. Various kinds of benchmarks were used, from the concrete pillars for the Lipari-Vulcano network, to the nails or reinforcing bars used on Etna. The first experimental GPS measurements were carried out on Etna in 1988 (ref. 2), and later GPS measurements progressively replaced the trilateration ones on all areas, while automated topographic measurements are currently only done for monitoring the Sciara del Fuoco at Stromboli^20^. The change in methodology also prompted a gradual re-configuration of all networks, due to the different requirements and capabilities of the GPS with respect to the optical instrumentation. Furthermore, nails or reinforcing bars were replaced by self-centering benchmarks in order to avoid the typical station setup errors when using tripods (Fig. 1). Self-centering benchmarks consist of brass cylinders that are permanently installed in solid outcrops or stable reinforced concrete structures by simply drilling a 5 cm wide hole and accurately fixing them with resin epoxy. The internal part of the cylinders are designed to perfectly contain and fix, without any movement, the bottom of the geodetic pins that are screwed into them during the measurements (see Fig. 1).

IIV started monitoring ground deformation on seismic areas after the December 13th, 1990 M 5.4 earthquake^21^, which struck the northern border of the Hyblean mountains (SE Sicily), when a first EDM network was installed around the epicentral area. This first attempt was the starting point for implementing a wider geodetic network covering the whole of eastern Sicily. The benchmarks of this network were pillars made in concrete or iron. In 1991, the former EDM network was expanded by using some these pillars and measured by using GPS^1^. A network of self-centering benchmarks was installed and measured by GPS in 1996 across the Tindari-Letoianni fault system, on the Peloritani Mts. (North Sicily^22^). In 2001, some pillars across the Messina straits were measured and later on, in 2006, the Mt. Peloritani and Messina Strait networks were measured together during a single survey.

The use of GPS to monitor ground deformation in Sicily had a dramatic and evident impact on the network’s geometry for three reasons: GPS does not need the inter-visibility of the measurement points, which was one of the main drawbacks of the oldest techniques (e.g. EDM); GPS offers a wide spectrum of surveying techniques (from static to kinematic ones) allowing researchers to adopt the most suitable one for the given environmental conditions, dynamic processes, etc.; lastly, the use of GPS receivers have become increasingly easier (e.g. the weight and power consumptions have been reduced, the field operations become more user-friendly, etc.). These effects are particularly evident on Etna, due to the re-location of some benchmarks to sites that are easier to reach. This is the case, for instance, of benchmarks previously installed on the top of hills for inter-visibility, which were re-installed at their bases allowing quicker station set-ups and for a better integration with other discrete geophysical measurements, such as microgravity ones, for more complete multi-disciplinary investigations^23^. Moreover, the initial network was radically expanded by installing some sub-networks on the eastern flank^24^ and across the Pernicana fault^25,26^. The semi-kinematic survey method, tested in the 90’s, enabled designing and installing dense cross-profiles, N-S and E-W oriented, thereby increasing the spatial detail in the ground deformation sampling on the upper part of the volcano, by setting-up tens of benchmarks aligned along profiles already surveyed by microgravity measurements^27,28^. From 2001 on, all the sub-networks on Mt. Etna were unified and surveyed in a single campaign^29^. The use of the double-frequency fast-static survey mode, allows a faster survey of the local network installed on the ‘La Fossa’ crater at Vulcano and the semi-kinematic technique is exploited for surveying its ‘La Forgia’ unstable sector^30,31^.

In a minor but still significant way, all geodetic networks have been and are continuously improved, by replacing the less precise, old and/or destroyed benchmarks with the new self-centering ones. The Hyblean network was thus first expanded^32^ and then improved^14^, as well as the networks at Pantelleria^33^, Peloritani—Messina Straits and Lipari-Vulcano^13,34^. All the networks contribute considerably towards a better understanding of the global and local geodynamics of eastern Sicily, providing further details of the crustal deformation and allowing more complex modeling of the volcanic dynamics and of the local to regional tectonics^1–4,6–15,23–30,32,33,35–41^, as well as for calibrating and constraining different geophysical investigations^42–44^.

### GPS surveys in Sicily

From June 1994 to June 2013, GPS surveys on Etna Mount, Aeolian Islands, Hyblean mountains, Pantelleria Island, and the Peloritani Mountains were routinely carried out. Only in 1999 were no complete surveys carried out and no useful data has been found in the archives for the database. The specific characteristics of the geodynamics of each area have guided the planning of the interval between surveys and the specific surveying technic adopted. For instance, campaigns are usually performed at least yearly on Mt. Etna, though more frequent observations (even daily or weekly) have been carried out during periods of high volcanic activity. Conversely, seismogenic areas (e.g. Hyblean or Peloritani mountains) as well as Pantelleria island, characterized by a slow continuous dynamic, have been surveyed only three-four times during the 20 years of this database. Data have been collected by different receivers: Trimble 4000 SSE, Trimble 4000SSI, Trimble 4700 and Trimble 5700 equipped by different accessories, such as Trimble TDC1 and TSC1 controllers, Trimble 41249.00, Trimble 14532.00 (with ground plane), Trimble 22020.00 (with ground plane), Trimble 29659.00, Trimble 33429.00 (with ground plane) and Leica AX1202GG antennas. The set-up of the antennas over the benchmarks was initially performed only either by screwing the antenna directly on the EDM pillars (in some cases using a steel base) or mounting the tripods over the nails; these kinds of station set-ups, especially those on tripods, have been replaced by fixed-height, self-centering benchmarks in order to increase the accuracy of either the position and the repeatability of the set-up.

Commonly used techniques during these surveys are static, fast-static and post-processed (PP) kinematic (stop&go). In static surveys, the most frequently used for the highest accuracy in the position, each receiver at each point logs data continuously for a pre-planned length of time with a 30-seconds acquisition frequency. The session duration was about 3–4 h during the first surveys, by measuring each benchmark at least 2 times; later on, the duration was increased up to 24 h sessions as the benchmarks were relocated in more secure places, by installing the GPS equipment in a semi-permanent way for at least a couple of days.

The fast-static method is usually adopted to survey areas where a high detail of the deformation pattern is needed, without increasing too much the duration of the entire survey, such as the Vulcano-North network around and on the La Fossa crater at Vulcano island^13^. This kind of measurement allows shorter observation times but with a lower accuracy. During this kind of survey, some receivers perform 20 to 30 min sessions (longer than the minimum time required and depending on the satellite constellation available during the measurement) on each point of the network, by surveying each benchmark twice in order to have two independent measurements and then two independent sets of coordinates for them with different satellites configurations. The acquisition rate for these sessions is increased to a 5 s frequency for all the stations acquiring contemporaneously during the survey. In the case of the Vulcano-North network, the steep morphology of the crater together with the conditions of the trails constrain surveying the network in one or two days by two or three teams walking on and around the crater.

In the PP kinematic stop&go method, some stationary receivers, called base stations, are placed at known points (which are measured also by static method during the same survey), while other receivers called ’rover’ consecutively visit all the benchmarks of the network. This method is used for measuring very dense networks of points aligned along a path (profile) that must be taken on foot (e.g. La Forgia network at Vulcano island^30^) or by car (e.g. the N-S profile^36^ and the E-W profile^27^ on Mt. Etna). The rover receiver has to be ‘initialized’ by carrying out a static measurement at the first station (usually lasting 1 h). The receiver then starts to move without interrupting the satellites tracking and data recording, performing 3 to 5 min stops at each benchmark of the surveyed profile. The survey is performed by adopting a back-and-forth strategy, i.e., after having measured the network in one direction, a longer measurement (usually 10 to 30 min) is performed at the end of the profile, before coming back, re-measuring all points with the same technique. The acquisition frequency is usually 5 s for all base and rover stations.

Clearly, the accuracy diminishes from the static to ‘fast-and-go’ technique; however, the speed up increases in the same direction, so the choice of one specific survey technique is made by taking into account the final expected accuracy (conditioned by the dynamics of the area) and the operational conditions (available instruments, number of people, cars, etc.).

Creating this database entailed five main steps: (1) collection and checking the raw GPS data; (2) collection of all supplementary data relevant to the available raw GPS data; (3) conversion of the raw data in RINEX format; (4) validation of the RINEX files and (5) the creation of the database.

During the first four steps, we had to deal with several problems related mainly to lost or incomplete data, broken files and lack of uniformity in acronyms or standards, which were solved by correlating information among different surveys or looking for backup copies of the data in different storage devices or processing machines.

### Data collection

Raw GPS data are saved as binary files in the receiver’s proprietary format. The files have usually been downloaded from the receivers in duplicate copies, on multiple physical supports, right after each survey. A partial collection and organization work was recently done and provided only for data collected on the Eolian islands networks^37^. Thus the first work was to look for the thousands of files archived over the years and check their readability. The main difficulty here was to recover files from old physical supports (e.g. 3.5’ disks); in some cases the possibility of having duplicate original downloads of the same file/s was fundamental to successfully recover the data. Thanks to this phase, we have verified that 100% of the original data have been successfully recovered from 1994. As said, 1999 is the only missing year, since no useful data were found in the archives. Unfortunately, most of the very old data collected before 1994, stored in 3.5’ floppy disks, were severely corrupted and we were not able to create RINEX files; so we decided to publish the most complete dataset, namely from 1994. Having verified that the specific file is still readable, it was archived in a NAS for successive processing. In order to facilitate the RINEX conversion, the collected data has been stored and organized in a structured archive.

### Metadata collection

**Figure 2:**
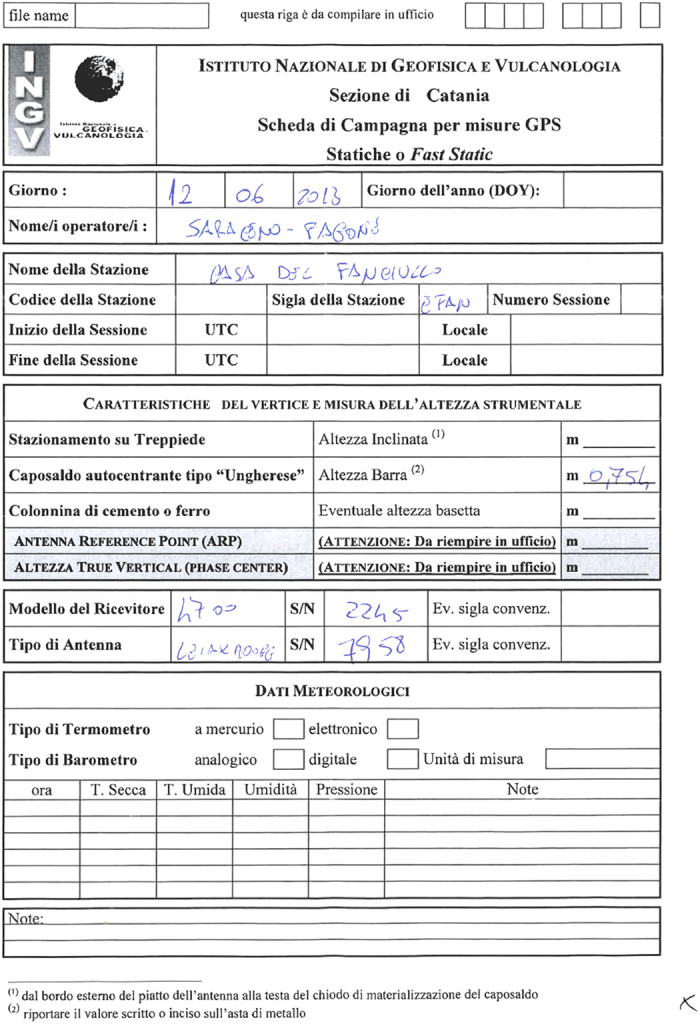
Scan of a typical survey paper sheet (station EFAN during the Etna June 2013 survey). The sheet contains the following information: DOY (Day Of Year) of the measurement, the site name, the site code, the session start/end, the operators, the receiver type, the receiver serial number, the antenna type, the antenna serial number, the type of benchmark or set up, the measured instrumental height, the calculated ARP (Antenna Reference Point) height, and the GPS week.

In order to convert raw data in RINEX file, it is first necessary to collect and organize all needed supplementary information. An Excel sheet was initially created for each survey, retrieving and summarizing all the available information from the original field paper sheets (Fig. 2) or information from old RINEX files prepared during previous analyses of these data. Rarely was information lost, though occasionally for some for very old surveys; we chose to produce and provide the files in any case and this further emphasizes the importance of this collection and the archiving work.

To ensure the consistency in the criteria to define the supplementary information, a few general rules for standardizing the information have been adopted, e.g. the uniformity of the names of the station or the surveys, networks, folders etc.

In the following, we illustrate the adopted criteria in detail.

The criteria to name each benchmark has changed over so many years. Moreover, sometimes benchmarks were relocated thus requiring a new name. For these reasons, a complete revision and validation of station names has been performed throughout the period covered by the GPS data to avoid ambiguities.

For most networks, station names start with a letter depending on the location; so, stations at Vulcano island start with a V, stations at Lipari island start with a L, stations at Pantelleria island start with a P, always followed by a three letter acronym for the the site name (e.g. VCRA: VulcanoCRAter, LFAL: Lipari FALcone, PMUE: PantelleriaMUEggen). Old Hyblean (Iblei, in Italian) stations start with IP (for iron pillars) or with IM (for concrete pillars, Masters) followed by a progressive number (e.g. IP09, IM01); new Hyblean benchmarks start with an I, for (e.g. ICAR: IbleiCARlentini). Stations on the Messina Straits start with MM followed by a progressive number (e.g. MM03). New benchmarks at Mt. Etna start with an E (e.g. EMIR: Etna MIRio).

Acronyms for the file name have also been standardized. Old benchmark acronyms had three characters (or even less), while the current standard is four characters. In the three-character cases, we added 0 (zeroes) at the end of the original acronym. For example, the old benchmark NUN (Mt. NUNziata) at Etna was renamed to NUN0 in this archive; recently, this benchmark was re-located by installing a self-centering one and renamed ENUN. Stations on the La Fossa crater of the Vulcano Nord Network, such as V1, S3 and so on, were renamed in this archive by adding two zeroes, becoming V100, S300 and so on. This renaming procedure was not necessary for the La Forgia benchmarks at Vulcano, since they were surveyed by kinematic mode and the station acronyms were not used to name the unique file containing all measurements; in particular, kinematic measurements files were named starting FRGA for the La Forgia profile, KINS for the Etna N-S profile and KIEW for the Etna E-W one. Acronyms of each station surveyed during a single stop&go measurement session are stored in the unique file produced during the measurement and can have only three characters since they do not influence the file naming.

During this collection and organization work, we had to tackle also many problems mainly related to lost or incomplete field sheets, broken files and no uniformity of data. These problems have been solved through research and correlation with similar data or other surveys, with old RINEX already created with other software and with standardization of all data. One recurring problem was the lack of or incomplete information about the antenna type on the field paper sheet; this was usually resolved by cross-correlating all measurements carried out at all stations during each session and each survey. In this way, all the available antennas were catalogued for the given survey and, cross-correlating all survey sheets for the given session, the correct antenna was deduced.

In case of power failure of the station during a measurement session, the raw data file in the receiver is not correctly closed. Data is not lost but the approximated and mean station position is not saved. This means the produced RINEX file will not have the correct ‘a priori’ approximated X, Y and Z Cartesian coordinates (ECEF: Earth Centered—Earth Fixed) in the header, reporting the default ‘0000000.0000 0000000.0000 0000000.0000’ values. This shortcoming could generate some problems in the processing with different software. To fix this, a list of all coordinates was generated for all stations surveyed during all the surveys considered in this work and correct approximated ‘a priori’ coordinates were manually copied into the RINEX files affected by this kind of error.

### RINEX conversion

RINEX (Receiver INdependent EXchange) is the ASCII standardized interchange format for any GNSS (Global Navigation Satellite System) data (Gurtner and Estey, 2007). Each RINEX file is self-consistent, i.e. it also contains all necessary metadata allowing the user to post-process the GNSS data for helping produce a very accurate solution.

**Figure 3:**
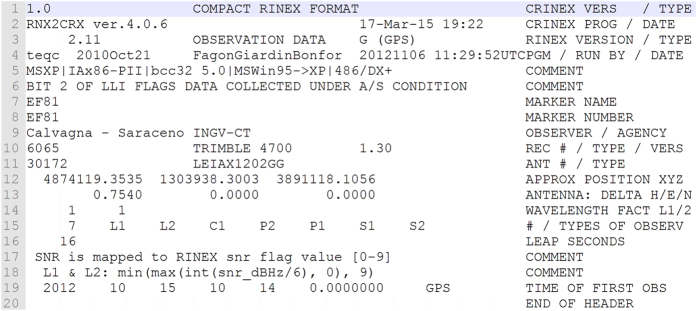
Example of the header of a RINEX 2.11 file. It contains all the supplementary information needed for an accurate processing of the GPS data. This example refers to a measurement session carried out on the EF81 benchmark, by using a Trimble 4700 receiver and a Leica 1202 antenna; the ARP is 0.754 m.

Starting from the previous collection of the data and metadata, RINEX files (2.11 format^5^) have been created by using one of the functions of the TEQC software^45^ (TEQC: The Multi-Purpose Toolkit for GPS/GLONASS Data, release 15-03-2013), which enables converting any binary raw GNSS data into a RINEX file by running an appropriately implemented script under Linux OS. Because the observables included in the RINEX data depend closely on the receiver used for the different surveys, we adopted the criteria to include all phases (L1 and L2) and codes (C1, P1 and P2) and signal-to-noise ratios (for both carriers, S1 and S2) in the files (Figure 3). RINEX 2.11 files were then compressed into the standard compact-RINEX format to save space^46^.

By adopting the standard, the RINEX file name is composed of eight characters (4+3+1): station acronym ($$$$, 4 characters), DOY (Day Of the Year, DDD, three numbers) and session number (S) for 30-second acquisition rates or a ‘K’ for 5 s (kinematic or fast-static sessions) acquisition rates. The file extension is the usual standard three-character extension of the RINEX format, i.e. two numbers indicating the year (YY) and a ‘d’ indicating that they are compressed observation files. In this way all station names have the standard structure $$$$DDDS.YYd (e.g. EMIR1890.10d, for station EMIR, Day Of the Year number 189, that is July 8th, static session, year 2010).

### Code availability

Software used to create the RINEX files and compress them represents the standard and are well established in the geodetic community. TEQC software package is available from UNAVCO as the standard tool for creating RINEX files and checking the quality of the measurements, under Linux and Windows environments^45^. GAMIT software package (it stands for GPS Analysis at MIT) runs under Linux OS and is developer and freely distributed by Massachusetts Institute of Technology (MIT)^47^. The Crx2rnx tool^46^ for compressing RINEX files is freely distributed by UNAVCO. Software for reading and exploring the geospatial database is based on the SharpMap open source mapping library (available on https://sharpmap.codeplex.com under GNU Lesser general Public License).

## Data Records

**Figure 4:**
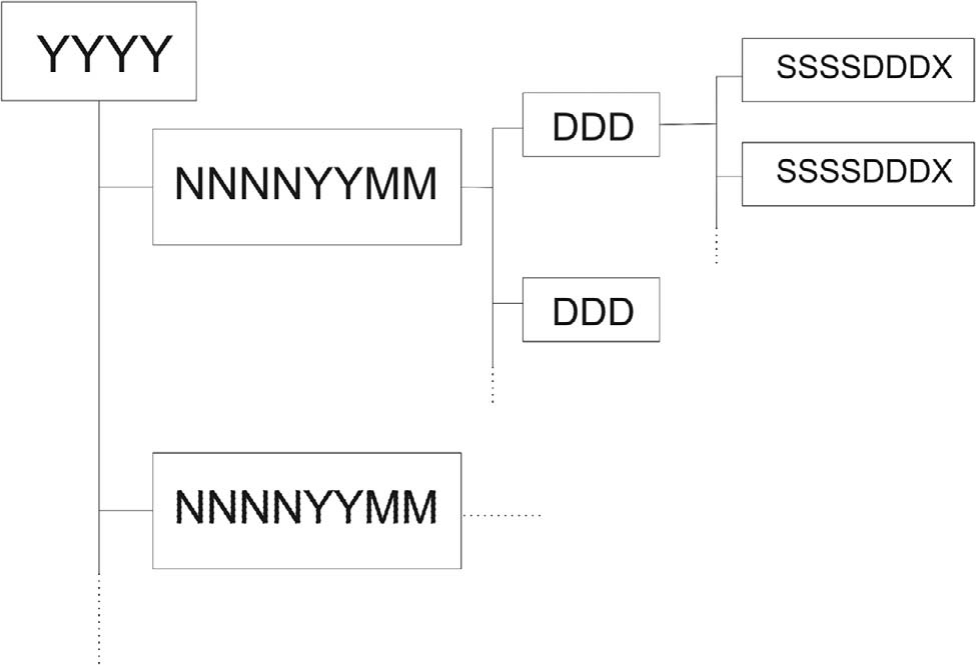
Schematic view of the Archive structure.

Once the raw data have been translated in RINEX files and then checked, the RINEX files have been deposited in an appropriately structured repository (Data Citation 1). The folders are organized by year, survey name and DOY. The structure of the archive tree is reported in Fig. 4.

The top level of the archive is the year. The folder name is simply made up of the four numbers of the year (YYYY). Inside each year folder, data are organized by survey. The survey folder names have eight characters indicating the acronym of the area (NNNN, first four characters) the year (YY, two characters) and the month (MM, last two characters); in this way there is a unique name for each survey, even in the same area. We used the following acronyms for the surveyed areas: ‘Etna’ for Mt. Etna, ‘Ioni’ for Ionica (eastern Etna), ‘Pern’ for Pernicana fault, ‘Livu’ for Lipari-Vulcano, ‘Pant’ for Pantelleria, ‘Mess’ for Messina Straits (recently including also Peloritani), ‘Hibl’ for Hyblean and ‘Pelo’ for Peloritani. Sometimes a number of stations were installed on some sites for experiments without surveying a specific area; we also provide these data, naming these surveys simply with the station names.

In total, more than 6200 files were processed for the entire 18-year period, as reported in Table 1 (available online only).

## Technical Validation

**Figure 5:**
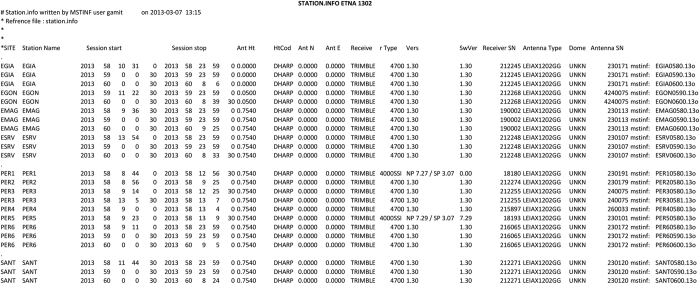
Example of a Station.info file (Etna February 2013 survey).

In order to validate the results of the translation processing, the information uploaded in the RINEX files were extracted by using a function of the GAMIT software^47^, which is often adopted to process GNSS data, for geodetic purposes. One of the GAMIT utilities, ‘upd_stinfo’ script, scans the previously created RINEX files and extracts the metadata by creating an ASCII file named ‘station.info’, which contains: site code, session start, session stop, antenna height, receiver type and its serial number, antenna type and its serial number and other minor information (Fig. 5). Each row is compiled by appending the metadata concerning the information of each measurement at each station.

A visual inspection of the ‘station.info’ make searching for any anomalous value/character easy, for instance, in the name of the antenna/receiver type, date, time, etc.

Each station.info file was renamed and uploaded in the database by using the following form: station_$$$$YYMM.info: station acronym ($$$$, 4 characters), two numbers indicating the year (YY), then two numbers indicating the month (MM). We provide these files in the archive, one for each survey, summarizing all the necessary supplementary information in an easier and more standard way than an excel sheet.

### Analysis of data structure and extraction of metadata

This section details our approach to implementing a data browser and query mask for extracting and managing the RINEX data of the archive provided.

As a first step, we proceeded by analyzing the RINEX content by rows, since from each one of the header rows we can gather some useful information for the end user (Fig. 3).

The alphanumeric information in each row is separated by a variable number of spaces, so it can be easily detected. Furthermore, in almost all cases, each piece of information is located at a fixed position, starting from a specified line and column. Starting from this, we implemented some scripts to automatically extract all the alphanumeric data for use as input for the database.

## Usage Notes

To manage the information using a GIS-approach, a geographical component has been added to the database. By exploiting the APPROX POSITION XYZ row in the file headers, the approximated station position is known and can be inserted into the database. Through appropriate transformations, the Cartesian geo-centric coordinates archived in the RINEX files are converted into E, N, H by assuming the WGS84 datum. By these coordinates, through a GIS software, we associated a point geometry to each file, creating a GeoDataBase, which allows the user to perform spatial queries and analyses. The fields of the final geographic database are reported in Table 2.

After compiling the geographic database, we proceeded to create a browser software, based on the SharpMap code; this browser performs the data search by different alphanumeric and geographic parameters and its visualization.

### The geographic database

**Figure 6:**
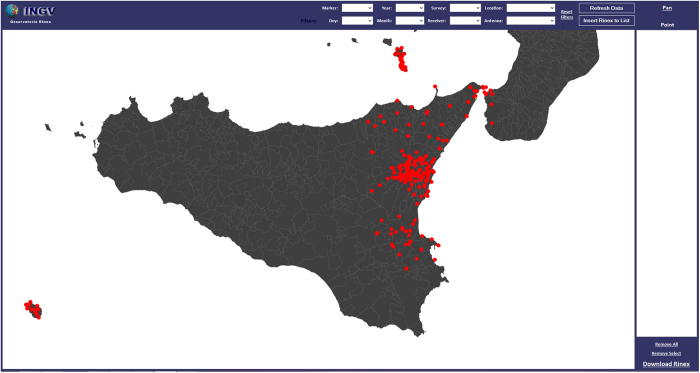
Software start screen, with all data visualized on the map.

When the software is run, a start screen is displayed as in Fig. 6. Two geometric features are displayed on the main screen: polygons for the administrative boundaries and points representing the RINEX data. At the top of the window, using the drop down menu, some filters can be applied to the dataset, based on some of the fields reported in Table 2. By selecting a filter and clicking the ‘refresh data’ button, only the data satisfying the specified conditions will be displayed.

**Figure 7:**
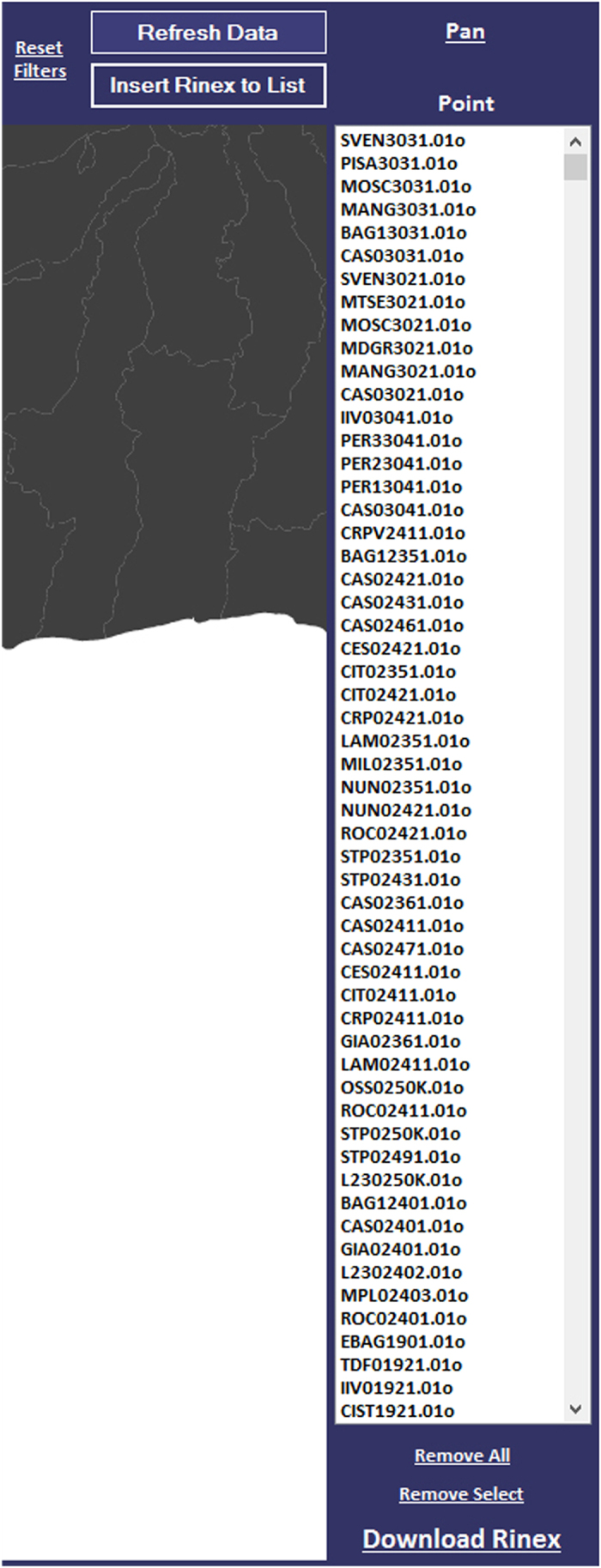
The section ‘Point’ on the right side of the screen.

After the search is completed and only the wanted data displayed, it is possible to download the filtered data by clicking on the ‘Insert Rinex to list’ button. The selected data will be listed in the section ‘Point’ on the right side of the window. Clicking ‘Download Rinex’ begins downloading the data from the archive (Fig. 7). It is also possible to remove single files from the list, by selecting the unwanted data and clicking on ‘Remove Select’. ‘Remove all’ clears the list for a new search.

**Figure 8:**
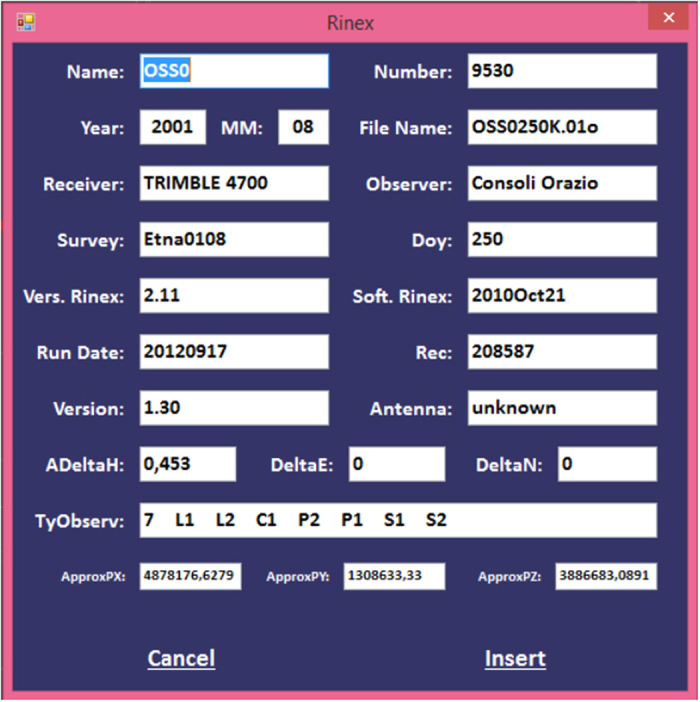
Dialog box with all the information about the selected Rinex file.

Clicking the Pan button at the top right of the window (Fig. 8) enables ‘Get Info’. It is then possible to gather information about the data through a pop-up window (Fig. 8) with a table showing all the fields of Table 2 by simply clicking on a point. To add the associated Rinex file, clicking ‘Insert’ adds the file to the list. Otherwise, the pop up window can be closed without any effect by clicking ‘cancel’.

## Additional Information

**How to cite this article**: Bonforte, A. *et al.* Global positioning system survey data for active seismic and volcanic areas of eastern Sicily, 1994 to 2013. *Sci. Data* 3:160062 doi: 10.1038/sdata.2016.62 (2016).

## Supplementary Material



## Figures and Tables

**Table 1:** Summary of the processed files for each survey and total

**YEAR**	**SURVEY**	**# of processed files**
**1994**	Etna9406	18
	Etna9407	65
	Etna9409	8
**1995**	Etna9503	26
	Etna9506	69
	Etna9510	54
	Etna9511	42
	Pelo9510	8
**1996**	Etna9607	90
	Etna9609	30
	Pelo9610	15
**1997**	Etna9704	6
	Etna9706	74
	Ioni9709	44
**1998**	Etna9801	39
	Etna9804	3
	Etna9807	86
	Etna9811	6
	Hibl9804	40
	IIV09805	10
	IIV09809	6
	Ioni9808	86
	Osv09801	10
	Pelo9811	30
	Pern9802	8
	Pern9806	9
	Pern9810	6
**2000**	Etna0007	88
	Hibl0005	88
	Pant0011	24
**2001**	Etna0106	144
	Etna0107	70
	Etna0108	148
	Etna0110	11
	IIV00104	54
	Ioni0101	42
	Ioni0110	27
	Isla0111	6
	Mess0102	24
	Pelo0106	6
	Pern0103	6
**2002**	Etna0204	5
	Etna0207	156
	Livu0205	62
	Pelo0209	14
	Pelo0210	81
	Pern0203	6
**2003**	Etna0306	204
	Etna0312	6
	Livu0304	63
	Pant0309	23
	Pelo0310	34
	Pelo0311	67
	Pern0302	68
	Pern0311	12
**2004**	Etna0401	16
	Etna0406	190
	Etna0409	17
	Livu0403	51
	Pern0401	11
	Pern0402	24
	Pern0403	89
	Pern0406	54
	Pern0410	11
	Vulc0412	43
**2005**	Etna0506	69
	Etna0507	176
	Etna0508	23
	Etna0511	15
	Hibl0511	28
	Livu0504	64
	Pern0504	11
	Pern0511	16
	Stmb0504	4
	Vulc0511	40
**2006**	Etna0606	205
	Etna0610	9
	Hibl0603	49
	Livu0603	49
	Mess0611	68
	Pern0604	10
	Pern0609	25
	Pern0610	6
**2007**	Etna0701	18
	Etna0704	10
	Etna0705	219
	Etna0711	11
	LiVu0709	69
**2008**	Etna0802	39
	Etna0804	18
	Etna0805	128
	Etna0806	194
	Etna0811	41
	Livu0810	68
	Mess0803	36
	Pant0804	44
**2009**	Etna0904	38
	Etna0905	8
	Etna0906	207
	Livu0910	74
**2010**	Etna1004	46
	Etna1006	175
	Etna1011	36
	Livu1009	72
**2011**	Etna1106	163
	LiVu1111	60
	Mess1107	49
**2012**	Etna1206	169
	Etna1210	79
	LiVu1209	79
	Pant1205	36
**2013**	Etna1302	23
	Etna1303	37
	Etna1306	190
	Vulc1304	108
	**TOTAL**	**6344**

**Table 2:** Fields of final geographic database.

DOY	OBSERVER
VERS_RINEX	SOFT_RINEX
RUN_DATE	RUN_TIME
REC	RECEIVER
VERSION	ANTENNA
A_DELTA_H	A_DELTA_E
A_DELTA_N	T_OBSERV
APPROX_P_X	APPROX_P_Y
APPROX_P_Z	GEOC_POS_X
GEOC_POS_Y	GEOC_POS_Z
PATH	
M_NAME	M_NUMBER
YEAR	MONTH
FILE_NAME	SURVEY
