# Corrigendum: Global positioning system survey data for active seismic and volcanic areas of eastern Sicily, 1994 to 2013

**DOI:** 10.1038/sdata.2016.77

**Published:** 2016-09-13

**Authors:** Alessandro Bonforte, Sonia Fagone, Carmelo Giardina, Simone Genovese, Gianpiero Aiesi, Francesco Calvagna, Massimo Cantarero, Orazio Consoli, Salvatore Consoli, Francesco Guglielmino, Biagio Puglisi, Giuseppe Puglisi, Benedetto Saraceno

**Keywords:** Geophysics, Natural hazards, Tectonics, Volcanology, Geodynamics

*Scientific Data* 3:160062 doi: 10.1038/sdata.2016.62 (2016); Published 1 August 2016; Updated 13 September 2016

The authors regret that financial support for this Data Descriptor was not fully acknowledged. The Acknowledgements should have read:

## Acknowledgments

The authors are indebted to P. Briole and G. Nunnari for their enthusiasm towards experimenting with GPS methodology on Mt. Etna since 1988 and for their work on making it a powerful tool for research and monitoring in volcanology. Sincere thanks are also due to Prof L. Villari for having started the ground deformation study on Sicilian volcanoes in the 1970s and for helping carry out the first GPS campaigns. Special thanks are due to M. Bonaccorso for his huge effort in building the initial GPS networks and carrying out the GPS measurements in the ‘90s. The authors wish to thank all the colleagues who participated in one or more of the huge number of measurements carried out to produce this unique database. Thanks to Mauro Amore for developing a routine for the automatic control and validation of the RINEX headers, to Antonio D’Agostino for his support during the 2013 survey and to Ivan Nicolosi for his help in compiling the software. Thanks are due to S. Conway for reviewing the manuscript for language and grammar. The work of S. Fagone and C. Giardina was supported by the Servizio Civile Nazionale ‘Terra in movimento: monitoraggio geodetico delle aree a rischio volcanic e sismico della Sicilia’ 2012/2013 Project. This work has been carried out in the framework of the European FP7-MED-SUV (MEDiterranean SUpersite Volcanoes) project, grant agreement 308665.

